# Biological Functions and Regulatory Mechanisms of Hypoxia-Inducible Factor-1α in Ischemic Stroke

**DOI:** 10.3389/fimmu.2021.801985

**Published:** 2021-12-13

**Authors:** Qianyan He, Yinzhong Ma, Jie Liu, Dianhui Zhang, Jiaxin Ren, Ruoyu Zhao, JunLei Chang, Zhen-Ni Guo, Yi Yang

**Affiliations:** ^1^ Department of Neurology, The First Hospital of Jilin University, Changchun, China; ^2^ Shenzhen Key Laboratory of Biomimetic Materials and Cellular Immunomodulation, Institute of Biomedicine and Biotechnology, Shenzhen Institute of Advanced Technology, Chinese Academy of Sciences, Shenzhen, China

**Keywords:** HIF-1α, ischemic stroke, hypoxia, neurovascular unit, neuroprotection, neuroinflammation

## Abstract

Ischemic stroke is caused by insufficient cerebrovascular blood and oxygen supply. It is a major contributor to death or disability worldwide and has become a heavy societal and clinical burden. To date, effective treatments for ischemic stroke are limited, and innovative therapeutic methods are urgently needed. Hypoxia inducible factor-1α (HIF-1α) is a sensitive regulator of oxygen homeostasis, and its expression is rapidly induced after hypoxia/ischemia. It plays an extensive role in the pathophysiology of stroke, including neuronal survival, neuroinflammation, angiogenesis, glucose metabolism, and blood brain barrier regulation. In addition, the spatiotemporal expression profile of HIF-1α in the brain shifts with the progression of ischemic stroke; this has led to contradictory findings regarding its function in previous studies. Therefore, unveiling the Janus face of HIF-1α and its target genes in different type of cells and exploring the role of HIF-1α in inflammatory responses after ischemia is of great importance for revealing the pathogenesis and identifying new therapeutic targets for ischemic stroke. Herein, we provide a succinct overview of the current approaches targeting HIF-1α and summarize novel findings concerning HIF-1α regulation in different types of cells within neurovascular units, including neurons, endothelial cells, astrocytes, and microglia, during the different stages of ischemic stroke. The current representative translational approaches focused on neuroprotection by targeting HIF-1α are also discussed.

## Introduction

As the most vulnerable organ in the body, the brain requires an adequate and timely supply of oxygen and energy. Insufficient blood supply triggers a series of pathological events, leading to extensive death of neural cells in the ischemic region and neurological deficits within several hours, ultimately causing ischemic stroke. Bioenergetic failure, mitochondrial dysfunction, neuroexcitatory toxicity, inflammation, neuronal apoptosis, and protein folding errors may occur after cerebral ischemia, causing ischemic brain tissue to rapidly develop into the penumbra until irreversible infarction occurs (1). Management focuses on immediate revascularization by endovascular thrombectomy and intravenous thrombosis administration, which can improve the neurological deficits; however, time is a critical factor in both approaches. The pathological mechanisms underlying ischemic stroke have been extensively studied, which has resulted in a series of strategies and advanced methods for ischemic stroke treatment, such as anti-oxidative stress, neuroinflammation management, and neuroprotection (2, 3), with certain therapeutic effects in preclinical animal stroke models. Unfortunately, most of these strategies have failed in clinical trials; thus, there is an urgent need for innovative strategies that target the initial pathological changes, because timely treatment is critical for the prognosis of stroke patients (4, 5).

Hypoxia inducible factor-1α (HIF-1α) is considered a pivotal regulator of oxygen homeostasis and strictly regulated by oxygen levels (6, 7). HIF-1α mediates an endogenous adaptive program by regulating multiple signaling pathways and targeting downstream genes under hypoxic conditions. These genes have been found to be related to the pathophysiology of numerous neurological diseases, particularly ischemic stroke. Moreover, HIF-1α is involved not only in hypoxia but also in acute inflammatory responses, and it has recently been considered an important signaling regulator of inflammation (8, 9). A recent clinical study showed increased serum concentrations of HIF-1α at 48 h after acute ischemic stroke, and this result was positively correlated with the infarct size (*P* < 0.001). Although further verification is needed, HIF-1α will hopefully serve as a predictor of stroke prognosis (10). Studies have shown that HIF-1α has neuroprotective effects on cerebral ischemia, while others have reported negative effects such as interruption of the blood-brain barrier (BBB) integrity after stroke (11–14). Such divergent effects of HIF-1α in ischemic stroke may be attributed to the diversity of downstream targets, which function in different cell types within the neurovascular unit (NVU). Previous studies have found that HIF-1α is expressed in not only neurons but also astrocytes, endothelial cells, and microglia, and that it exerts cell type-specific actions (12, 15, 16). The function of neuronal HIF-1α has long attracted the interest of researchers, but the role of HIF-1α in other components of the NVU has received less attention.

In the present review, we elaborate on the regulatory mechanisms of HIF-1α and its various roles in ischemic stroke, specifically in four main types of cells in the NVU (i.e., neurons, astrocytes, endothelial cells, and microglial cells). Recent representative translational studies that propose novel therapeutic options targeting HIF-1α for ischemic stroke are also discussed.

## Structure of HIF-1α and Expression After Ischemic Stroke

**Figure 1:**
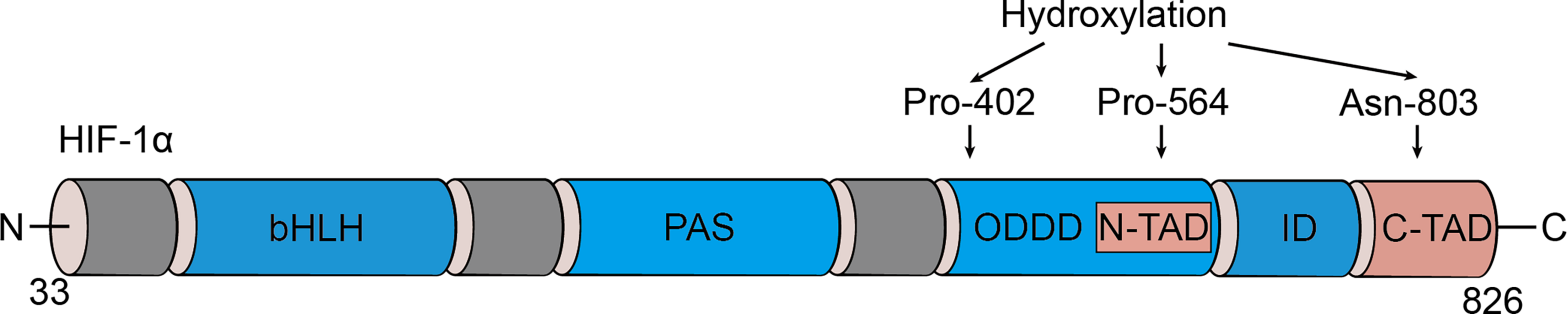
Domain structure of HIF-1α. HIF-1α is comprised of several conserved domains including a DNA binding (basic helix-loop-helix, bHLH) domain, protein/protein interactions and dimerization (PAS) domain, C-terminal trans-activation domain (C-TAD), N-terminal trans-activation domain (N-TAD), oxygen-dependent degradation domain (ODDD), and inhibitory domain (ID).

**Figure 2:**
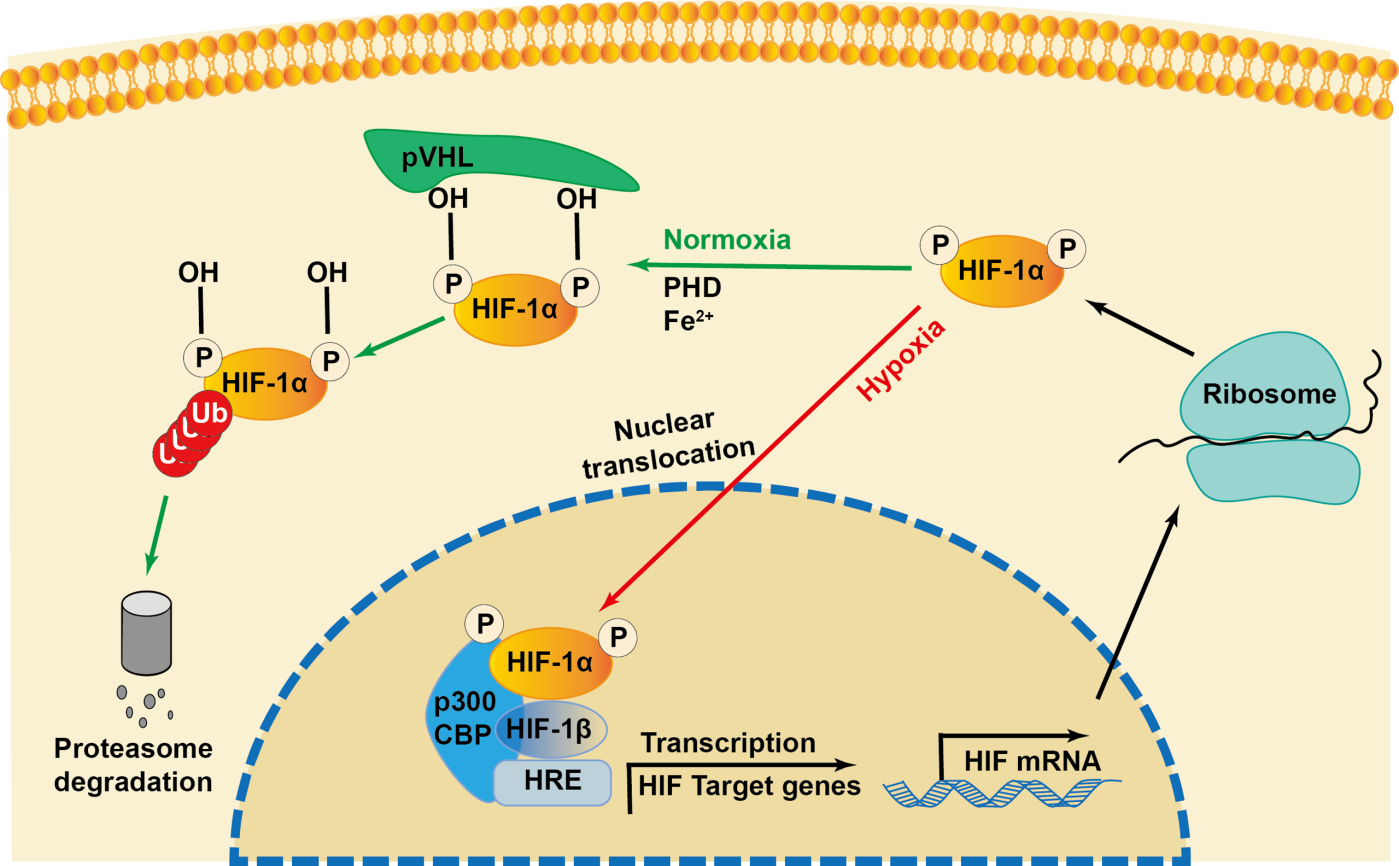
Regulation of HIF-1α during normoxia and hypoxia. Under normoxia, HIF-1α hydroxylase (PHD) hydroxylates the proline in the presence of iron (Fe2+), which recruits the von Hippel-Lindau protein (pVHL) to bind and initiate the proteolysis of HIF-1α by acting as a recognition component of ubiquitin ligase complex, leading to proteasomal degradation. Under hypoxia conditions, the interaction of transcriptional coactivators p300/CBP (CREB-binding protein) is activated. HIF-1α is stabilized and translocated into the nucleus and heterodimerizes with constitutively expressed HIF-1β, binds to the hypoxia responsive elements (HRE) and enhances the transcription of HIF-1α target genes.

Initially identified as a transcription factor, HIF-1, the prime regulator of oxygen homeostasis is a stable component of cells that can be activated and induced by decreased oxygen tension (17, 18). Once HIF-1 is activated, the transcription of genes related to adaptation and survival can be promoted. As a basic-helix-loop-helix-PAS heterodimer, HIF-1 comprises α and β subunits. HIF-1β heterodimerizes with other basic-helix-loop-helix-PAS proteins and is overexpressed in cells; thus, HIF-1α protein levels predominantly govern the transcriptional activity of HIF-1 (19). HIF-1α has an N-terminal transactivation domain, an oxygen-dependent degradation domain, a C-terminal transactivation domain, and an inhibitory domain (**Figure 1**). Under normoxic conditions, the key residues of proline (Pro-402 and Pro-564) in the HIF-1α subunit can be hydroxylated by prolyl hydroxylase (PHD) in response to 2-oxoglutrate and iron. After hydroxylation, the HIF-1α subunit is subsequently ubiquitinated by E3 ubiquitin ligase complex, which contains a Von Hippel–Lindau protein, a negative regulator of *HIF-1α* transcriptional activity, and is degraded by the 26S protein (20–23). Asparagine (Asn-803) is hydroxylated by asparagine hydroxylase, which inhibits its trans-activation ability and prevents HIF-1α from binding with CBP/p300 (a transcriptional coactivator) (22). When the cellular oxygen level decreases and PHD activity is inhibited, the asparagine and prolyl in the HIF-1α protein cannot be hydroxylated, resulting in the accumulation rather than ubiquitination of HIF-1α in the cytoplasm (24, 25). The stable HIF-1α forms a heterodimer with the HIF-1β subunit after translocating into the nucleus and binds to the hypoxia response elements on the promoter of HIF-1 target genes. This stimulates and enhances the transcription of the HIF-1 target gene, triggering the *in vivo* steady-state response of cells to oxygen and glucose deprivation (**Figure 2**) (21, 26). In addition to protein hydroxylation, the activity of HIF-1α is regulated by other post-translational modifications, including phosphorylation, acetylation, S-nitrosation, and small-ubiquitin-like modifier acylation (27).

The expression level of HIF-1α induced by stroke is related to the ischemic duration. In 1996, researchers first reported that both the HIF-1α and HIF-1β subunits were increased in rodents exposed to hypoxia for 60 min (28). Subsequently, the mRNA levels of *HIF-1α* and its downstream genes were found to be upregulated in the peri-infarct region at 7.5 h; they peaked at 24 h and declined at 72 h after the onset of focal cerebral ischemia in rats (29, 30). In another ischemia-reperfusion rat model, HIF-1α protein was induced at 4 h, peaked at 8 h, and declined at 24 h after reperfusion (31). The function of HIF-1α in ischemic stroke is bidirectional (**Table 1**). HIF-1α mediates the expression of a variety of genes that are involved in neurogenesis, angiogenesis, cell proliferation, erythropoiesis, and cellular metabolism. HIF-1α also participates in cell death, adaptive response, and cell regeneration, increasing its adaptation to ischemic stress, and consequently showing a neuroprotective role (60, 61). Conversely, several studies have reported the pernicious roles of HIF-1α in cerebral ischemic injury, including a severe inflammatory response, enhanced apoptosis, and loss of BBB integrity after ischemic stroke; this indicates that HIF-1α is likely a mediator of neuroinflammation or a factor determining the BBB permeability (62–64). Moreover, according to mounting data, HIF-1α appears to play a controversial role in cell autophagy (65–67). Taken together, HIF-1α increases after cerebral ischemia, and its expression level is dependent on ischemic duration, which may partially explain the seemingly contradictory effects on the pathological process of stroke.

**Table 1:** Bidirectional roles of HIF-1α in different cells.

Cell type	Neuroprotective effects	Detrimental effects
Neurons	Improve stroke related assessments *via* the upregulation of VEGF at 24 h after cerebral ischemia (32–37).	Aggravate BBB leakage as well as other stroke indicators *via* the upregulation of VEGF within 1 h after cerebral ischemia (32–37).
	Enhance erythropoiesis to fortify oxygen delivery and increase cerebral blood flow *via* the upregulation of EPO transcription in neurons (13, 38–40).	Increase necrotizing apoptosis through the RIP3/MLKL and NOTCH pathways (41, 42).
	Enhance the uptake of glucose *via* the upregulation of GLUT (14, 43, 44).	
	Increase the expression of NCX1 to facilitate cellular ionic equilibrium (45).	
Endothelial cells	Promote angiogenesis and neovascularization in ischemia-injured tissue by upregulating VEGF (30).	Exacerbate BBB permeability *via* increased VEGF (12, 46, 47).
Astrocytes	Provide protection against glutamate-induced excitatory toxicity (48, 49).	Exacerbate BBB permeability *via* increased VEGF (50–52).
	Facilitate glucose influx into astrocyte *via* the upregulation of GLUT1 thereby maintaining ATP levels and cell survival (53, 54).	Participate in neuroinflammation by promoting the secretion of chemokines (55).
Microglia	Induce the activation of autophagy in microglia and increase neuronal cellular viability (16, 56).	Promote microglia polarization, mediate the expression of proinflammatory factors and inflammatory responses, and aggravate neuronal damage by increasing the expression of TLR4 (57, 58).
		Activate NLRP3 in microglia and aggravate inflammatory responses (59).

HIF-1α, hypoxia inducible factor-1α; VEGF, vascular endothelial growth factor; EPO, erythropoietin; RIP3, receptor-interacting protein-3; MLKL, mixed lineage kinase domain-like protein; NCX1, neuronal sodium-calcium exchanger 1; GLUT 1, glucose transporters 1; BBB, blood brain barrier; ATP, adenosine triphosphate.

## Functions of HIF-1α in Different NVU Cells After Ischemic Stroke

**Figure 3:**
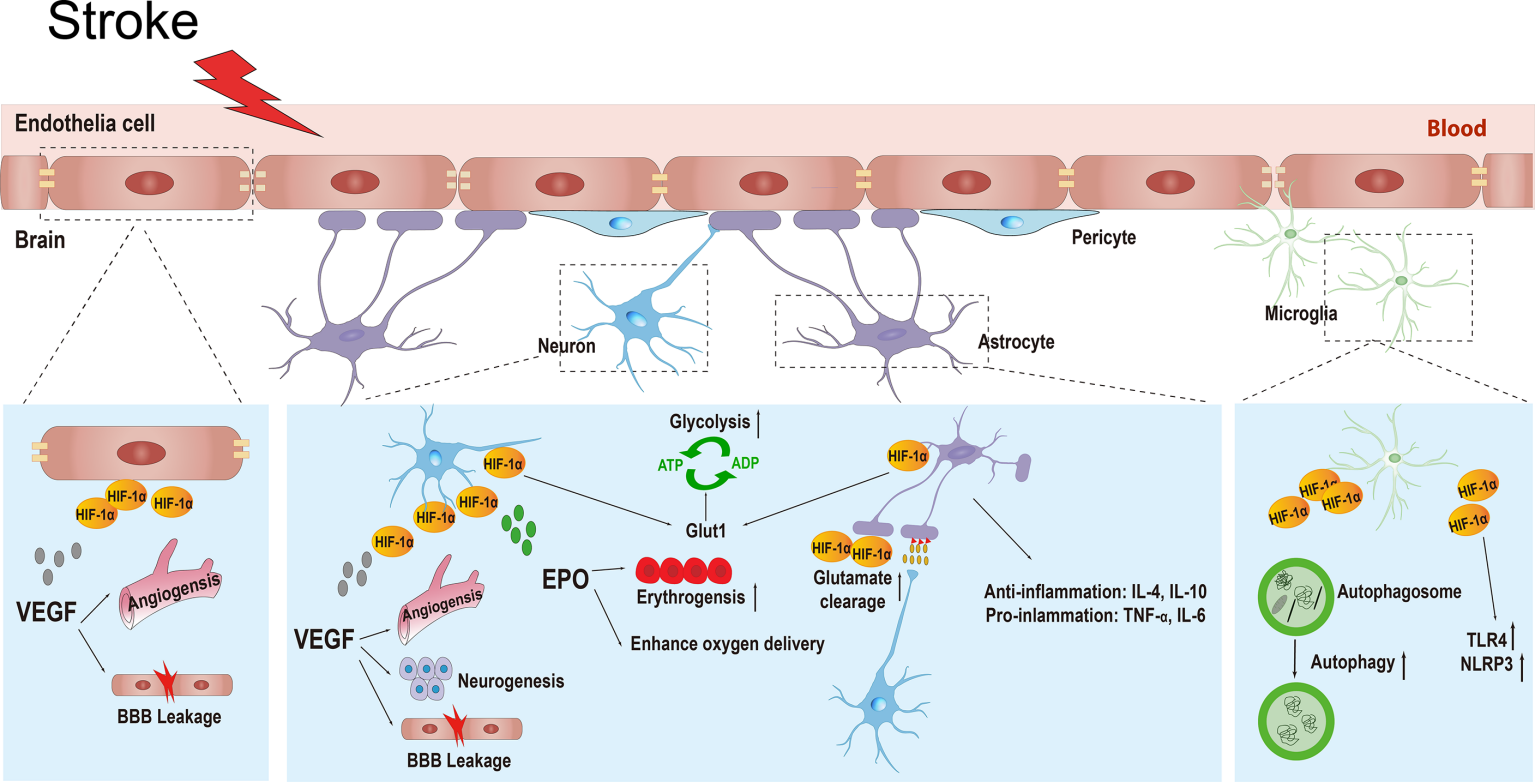
The various physiological functions of HIF-1α and its target genes in different types of cells in the neurovascular unit including neurons, endothelial cells, astrocytes, and microglial cells during ischemic stroke. HIF-1α, hypoxia inducible factor-1α; VEGF, vascular endothelial growth factor; BBB, blood brain barrier; EPO, erythropoietin; GLUT1, glucose transporter 1; ATP, adenosine-triphosphate; ADP, adenosine-diphosphate; IL-4, interleukin-4; IL-10, interleikin-10; TNF-α, tumor necrosis factor-α; IL-6, interleukin-6; TLR4, toll-like receptor 4; NLRP3, recombinant NLR Family, pyrin domain containing protein 3.

The definition of the NVU emphasizes the complexity of interactions between all perivascular components, including the basal membrane, neurons, astrocytic foot processes, pericytes, macrophages, and other leukocytes, and it integrates the contemporary concepts of the BBB (68). The central nervous system (CNS) relies on the co-operation of various types of cells and the ability of the BBB to function properly (69). During ischemic stroke, the BBB can be damaged both functionally and structurally by massive surrounding free radicals, calcium overload, ion imbalance, and autophagy, leading to brain edema or hemorrhagic transformation (70, 71). HIF-1α is widely expressed in the NVU and exhibits distinct functions, such as neuroprotection, BBB permeability regulation, angiogenesis, and inflammatory regulation (18, 61, 72). Till date, research has found that HIF-1α mediates the transcriptional expression of more than 100 genes under hypoxic conditions, and this number continues to increase (73). These HIF-1α target genes are involved in numerous processes, including cell metabolism, proliferation, survival, and death; cytoskeletal structure formation; cell adhesion; and movement. Because HIF-1α has various physiological functions in different types of cells, its phenotype becomes more complex under the context of ischemic stroke. In this article, we introduce the functions of HIF-1α and its downstream target genes in different cells in the NVU during stroke (**Figure 3**).

### Neurons

Neurons have been compared to pacemakers in the NVU and have a central role in the physiology of the CNS (74). Because of their high energy requirements, neurons are extremely sensitive to glucose and oxygen deficiencies (75), consequently, they are the most susceptible to ischemic injuries and die in large numbers within hours of hypoxia/ischemia. The pathophysiological function of HIF-1α in neurons varies, with contradictory effects on ischemic stroke.

#### Roles of Vascular Endothelial Growth Factor (VEGF) Regulated by HIF-1α in Neurons

VEGF, a well-known downstream target gene of HIF-1α, is extensively involved in the pathological processes of cerebral ischemia. The family of VEGFs is characterized by their strong angiogenic properties. In the ischemic brain, VEGFs are also important regulators of neuroprotection and neurogenesis (76). The neuroprotective effect of VEGF is thought to be mediated by the regulation of neurogenesis and angiogenic actions in ischemic brains at 12 h, but not immediately (i.e., 1 h), after stroke. In middle cerebral artery occlusion (MCAO) rat models, intracerebroventricular or intravenous administration of VEGF at 1 day or 2 days, respectively, after MCAO could reduce the infarction volume, improve neurological deficits, enhance the delayed survival of newborn neurons, and stimulate angiogenesis in the ischemic penumbra (32). Conversely, administration of VEGF 1 h after ischemia significantly exacerbates the neurological deficits (32, 33). In addition to angiogenesis and neurogenesis, the impact of VEGF is mediated through the control of apoptotic factors in ischemic brain tissues. Administration of HIF-1α-siRNA significantly reduces the content of p53 and caspase-3 and is accompanied by decreased VEGF expression, thus alleviating stroke-related behavior disorders and cerebral infarction at 1 h after MCAO (34). In another experimental rodent model of MCAO, 2-methoxyestradiol (2ME2; a HIF-1α inhibitor) therapy at 30 min after MCAO significantly decreased infarction size and the number of apoptotic neurons; however, 2ME2 delivered at 8 h after MCAO did not have a protective effect. Consistent with *in vivo* results, 2ME2 or HIF-1α-siRNA robustly reduced the expression of VEGF with aggravated apoptosis in neurons at 12 h after OGD treatment (35). Pretreatment with HIF-1α siRNA or treatment with HIF-1α inhibitor at 10 min after MCAO was found to alleviate BBB damage by reducing the *VEGF* mRNA level, alleviating occludin degradation, and preventing the excretion of matrix metalloproteinase (MMP)-2 in neurons (36). In general, activation of HIF-1α is deleterious immediately (i.e., 1 h) after acute ischemic stroke; however, after 12 h, it can show neuroprotective effects *via* regulation of VEGF-mediated angiogenesis and neurogenesis.

#### Roles of Erythropoietin (EPO) Regulated by HIF-1α in Neurons

EPO, another well-known target gene of HIF-1α, has been shown to protect cells from hypoxia injury by enhancing erythropoiesis to fortify oxygen delivery and increase cerebral blood flow. Administration of deferrioxamine (an iron chelator), both *in vivo* and *in vitro*, has been found to promote the expression of HIF-1α and EPO, thus improving the tolerance against cerebral ischemia, increasing neuronal viability, and exerting protective effects on OGD-cultured neurons and MCAO rats (38). A similar study found that activation of the HIF-1α/EPO signaling pathway in HT-22 cell lines facilitated pyruvate-mediated cytoprotection (39). Hypoxic preconditioning for 180 or 300 min can lead to the production of reactive oxygen species (ROS) and induce the production threshold of HIF-1α and EPO under ischemia, thus having a neuroprotective role through the NF-κ B/JAK2-tgf5 pathway (40). In a study conducted by Li et al., rats treated with Ad-HIF-1α 1 h after transient MCAO showed reduced neuronal apoptosis on day 7, which was attributed in part to the upregulation of EPO and the suppression of active caspase-3 (13). Therefore, the increased expression of EPO regulated by HIF-1α could provide a neuronal protective effect by increasing erythropoiesis to improve oxygen delivery during ischemic brain injury.

#### Regulation of Energy Metabolism by HIF-1α in Neurons

Energy metabolism is regulated by HIF-1α in neurons. The brain is a vulnerable organ that requires optimal delivery of oxygen and nutrients derived from blood. To meet the metabolic needs, glucose transporters (GLUT 1) are expressed in both endothelial cells and neurons to maintain the transportation of glucose. After ischemia, the enhanced nutrient demand by neural cells in the penumbra increases the need of nutrient transport across the endothelium. The activation of HIF-1α is conducive to cell survival by promoting glucose transport activity and glycolysis and maintaining redox equilibrium (43). In OGD-cultured SH-SY5Y cells, knockdown of *HIF-1α* hindered important components of the cellular redox equilibrium and critical enzymes of the pentose phosphate pathway and glucose metabolism, including glucose transporters such as GLUT1 (14). Research supports that hypoxia preconditioning can alleviate the loss of neurons in the rat cortex with traumatic brain injury (44); this mechanism is primarily associated with the upregulation of HIF-1α, which induces the expression of GLUT1 and GLUT3 and ultimately increases the uptake of glucose in the neurons.

#### Other Target Genes and Signaling Pathways Related to HIF-1α in Neurons

Other hypoxia-mediated signaling pathways related to HIF-1α have been identified to have a neuroprotective role after cerebral ischemia. PHD is a crucial mediator for the degradation process of HIF-1α, and inhibition of PHD activity could prevent the degradation of HIF-1α. Abundant experimental evidence has confirmed that the application of PHD inhibitors or *PHD* knockout may stimulate a protective cellular response in ischemic brain injury through the stabilization and activation of HIF-1α (77–79) (**Table 2**). In addition, the β-catenin signaling pathway has been shown to be related to HIF-1α. In a rat model of MCAO, a 42-d treatment with delayed hyperbaric oxygen was found to accelerate neurogenesis and improve neurological impairments, and those benefits could be counteracted by inhibiting HIF-1α and ROS (90). In response to hypoxia, upregulated HIF-1α could facilitate the level of neuronal sodium-calcium exchanger 1 (NCX1), the vital mediator maintaining the balance between sodium and calcium to sustain the stability of the intracellular environment. Valsecchi et al., revealed that HIF-1α can alleviate cerebral infarction by the upregulation of NCX1 in MCAO rats subjected to ischemia preconditioning and in OGD-cultured neurons, indicating that *NCX1* may be a new HIF-1α target gene (45).

**Table 2:** Therapeutic drugs that promote neuroprotection by regulating HIF-1α.

Therapeutic Drugs	Stroke model	Functions	Molecular Mechanisms	Reference
**PHD inhibitors**	OGD/MCAO	Repress neuronal apoptosis and cerebral infarction	↑ HIF-1α/EPO/VEGF/GLUT1	(80–82)
**Empagliflozin**	MCAO	Reduces neuronal death, ameliorate neurological disorders	↑ HIF-1α/VEGF ↓Caspase 3	(37)
**Tanshinone IIA**	OGD	Inhibits astrocyte proliferation	↓HIF-1α/SDF-1 signaling ↓ERK1/2/Akt signaling	(83)
**Partridgeberry polyphenols**	OGD	Reduces neuronal damage and sustain cellular viability	↓HIF-1α, TNF-α, IL-6 ↑PPAR-γ	(84)
**Berberine**	OGD/MCAO	Promotes cell survival and inhibits apoptosis	↓HIF-1α, Caspase 3 ↑Bcl-2/Bax ratio	(85)
**Ginkgolide K**	OGD/MCAO	Attenuates neurological deficits and promote angiogenesis	↑JAK2/STAT signaling ↑HIF-1α/VEGF	(86)
**Minocycline**	Hypoxia condition	Reduces BBB permeability and suppresses ROS generation	↓HIF-1α, PHD-2, SIRT-3	(87)
**Fluoxetine**	MCAO	Promotes angiogenesis and improves neurological function recovery	↑HIF-1α, Netrin/VEGF	(88)
**Valproate**	MCAO	Reduces brain infarction, enhances microvessel density, facilities EC proliferation and increases cerebral blood flow	↑HIF-1α, VEGF, MMP2/9 ↑Acetylation of histone-H3 and H4	(89)

PHD, prolyl hydroxylase; EPO, erythropoietin; OGD, oxygen glucose deprivation; MCAO, middle cerebral artery occlusion; SD rats, Sprague-Dawley rats; HBMECs, human brain microvascular endothelial cells; BBB, blood brain barrier; ROS, reactive oxygen species; EC, endothelial cells; HIF-1α, hypoxia inducible factor-1α; SDF-1, stromal cell-derived factor-1; ERK, extracellular regulated protein kinase; Akt, protein kinase B; TNF-α, tumor necrosis factor-α; IL-6:interleukin 6; PPAR-γ, peroxisome proliferators-activated receptor γ; Bcl-2, B-cell lymphoma 2; Bax, Bcl-2 associated X protein; JAK2, Janus kinase 2; STAT, signal transducer and activator of transcription; VEGF, vascular endothelial growth factor; PHD-2, prolyl hydroxylase 2; SIRT-3, sirtuin 3.

In contrast to its neuroprotective effects, HIF-1α has also been shown to be involved in some pro-necrotizing apoptosis signals and interact with the Notch signaling pathway under hypoxic conditions. The Notch intracellular domain (NICD) and HIF-1α are colocalized in the neuronal nucleus in brain tissue of MCAO mice. Overexpression of NICD and HIF-1α could aggravate cell death in OGD neuronal cell lines, where administration of 2ME2 reverses the cell death process. Furthermore, depletion of endogenous NICD and HIF-1α mediated by RNA interference were found to reduce cell death under hypoxic and ischemic conditions. Administration of HIF-1α inhibitors and γ-secretase in mice subjected to MCAO ameliorates neurological outcomes, and combination therapy was found to be superior to monotherapy (41). More recently, a study found that the simultaneous elevation of HIF-1α, mixed lineage kinase domain-like protein (MLKL), and receptor-interacting protein 3 (RIP3) in OGD HT-22 neuron cells are thought to participate in the formation and activation of the necrosome and have a significant role in necroptotic signaling. Therefore, for the first time, researchers proposed that HIF-1α is related to the occurrence of necrotizing apoptosis and may participate in ischemic brain injury and neuronal apoptosis by activating the RIP3/MLKL pathway (42).

### Endothelial Cells (ECs)

Brain ECs are the main components of the BBB. They form the wall of blood vessels and are recognized as the interface of blood–CNS exchange. The properties of the BBB are largely manifested within the ECs, whose stability is essential for maintaining the integrity of the BBB (91). The proliferation and migration of endothelial cells are largely dependent on VEGF. Beyond that, VEGF also increases vascular permeability, initiating the angiogenesis through the above properties (69). After stroke, the formation of new vessels is vital, as newly formed blood networks compensate the occluded vessel and thereby rescue the penumbra. However, the degradation of the vascular basement membrane during the activation phase in angiogenesis may lead to brain edema or hemorrhagic transformation in the acute phase of stroke.

#### Role of VEGF Regulated by HIF-1α in ECs

Compelling evidence has suggested that VEGF is robustly induced *via* the activation of HIF-1α after cerebral ischemic injury (92), and the functions of VEGF in endothelial cells are also bifurcated. As one of the most effective cytokines promoting the growth of vascular endothelial, VEGF stimulates the proliferation of ECs and directly participates in angiogenesis and neovascularization in ischemia-injured tissue. In a permanent MCAO mouse model, a substantial increase of newly formed vessels was observed at the ischemia penumbra 48–72 h after the occlusion, which was related to the robust increase of VEGF and VEGF receptors (VEGFR) mediated by HIF-1 and HIF-2, suggesting the regulatory function of HIFs in the VEGF/VEGFR system during the acute phase of stroke (30). Inevitably, in the acute phase of ischemic stroke, VEGF secreted from a variety of cell types increases capillary permeability to undergo endothelium-mesenchymal transformation, which causes BBB disruption (93).

In a rat cerebral endothelial cell hypoxia-reoxygenation transwell BBB model, the accumulation of VEGF and HIF-1α robustly increased the BBB permeability, in which the effect can be antagonized by HIF-1α inhibitors (94, 95). Consistent with the aforementioned study, hypoxic exposure stabilized HIF-1α rapidly, which is concomitant with VEGF secretion. As a consequence, tight junction proteins undergo increased tyrosine-phosphorylation and delocalization, ultimately leading to the loss of BBB integrity. Moreover, accumulation or stabilization of HIF-1α can also be achieved by using deferrioxamine, which further emphasizes the interaction of HIF-1α in BBB dysfunction, especially *via* tight junction alterations in hypoxic conditions (12). Administration of HIF-1α inhibitor or siRNA prior to MCAO surgery significantly suppressed the degradation of tight junction proteins and the upregulation of VEGF, ultimately alleviating BBB damage (36). In addition to VEGF, MMPs are known for their ability to regulate the BBB permeability. In a stroke rat model, administration of 2ME2 was found to reduce the expression of VEGF and the elevation of active MMPs 2 and 9, thereby attenuating hemorrhagic transformation and ameliorating neurological deficits in acute hyperglycemia-induced hemorrhagic transformation (46).

BBB impairment is particularly relevant in stroke patients with diabetes mellitus. Diabetes mellitus is an established risk factor for stroke that increases the absolute risk of stroke by about 1.5 to 3 fold (96–98), and diabetic patients with stroke are predisposed to worse outcomes (99, 100). Diabetes increases the risk of cerebrovascular accidents by altering the structure and function of arteries, arterioles, and capillaries. Over time, blood vessels become increasingly stiff, tortuous, and narrowed because of diabetes. The pathological processes of atherosclerosis and thrombogenesis are accelerated, which compromises the BBB permeability (101). Studies have shown that hyperglycemia can significantly upregulate the expression of HIF-1α and its target gene VEGF in the brain microvasculature after MCAO; this further aggravates BBB disruption after ischemic brain injury. Endothelial-specific HIF-1α knockout diabetic mice showed reduced BBB disruption and decreased brain infarction. In addition, diabetic mice treated with long-term insulin showed similar HIF-1α levels in the contralateral and ipsilateral hemispheres of the brain in stroke rats (47). Therefore, the accumulation of HIF-1α caused by hyperglycemia substantially aggravates BBB damage in diabetic stroke, and therapeutic treatments targeting HIF-1α may provide new treatment options for preventing hemorrhagic transformation in these patients.

#### HIF-1α and VEGF in Plaque Angiogenesis

Thromboembolism is the main cause of atherosclerotic stroke and is most frequently the result of plaque rupture, which is related to angiogenesis in the carotid plaque. Studies have shown that HIF-1α is relevant to plaque angiogenesis and can lead to plaque progression, bleeding, and ulceration (102, 103). In 2019, a clinical trial enrolled 54 patients who underwent carotid endarterectomy for severe internal carotid artery stenosis, of whom 20 patients were symptomatic and 34 were asymptomatic. The results of that study indicated that the serum mRNA levels of HIF-1α in carotid plaque endothelial and smooth muscle cells in symptomatic patients were 1.5-fold higher than those in asymptomatic patients, although no significant difference was observed between the two groups. Notably, the mRNA levels of *VEGF* were seven times higher in symptomatic patients than in asymptomatic patients (*P* = 0.017), indicating a correlation between VEGF and neurological deficits symptoms in patients with symptomatic carotid artery stenosis (104)

### Astrocytes

Astrocytes are critical components of the BBB, as they provide support and separate neural cells by stretching their cell bodies. As important active components in the NVU, astrocytes participate in two-way communication with other cells and have multiple roles during hypoxia and ischemia, including the release of cytokines, neurotrophic factors and nutrients to support neuronal survival (105–108). Notably, astrocytes mediate dual immunoregulation in ischemic stroke *via* the secretion of both proinflammatory and anti-inflammatory cytokines in response to changes in the cellular microenvironment, and the activation of HIF-1α may have a crucial role in regulating inflammatory responses (109, 110).

#### Roles of VEGF Regulated by HIF-1α in Astrocytes

As the core components of the BBB in the NVU, astrocytes have also been found to express HIF-1α, which may affect the integrity of the BBB. In OGD-cultured primary astrocytes, HIF-1α was found to be partly involved in the VEGF-mediated astrocyte response in chronic ischemic brain injury, ultimately leading to the destruction of BBB integrity. Preventing the action of HIF-1α downregulates the expression of VEGF, which not only attenuates the proliferation of primary astrocytes but also improves the integrity of the BBB (50). Notably, sirtuin 3 (SIRT3; known as the regulator of metabolism and aging) regulates VEGF expression by inhibiting HIF-1α signaling after permanent MCAO in rats or OGD in cultured primary astrocytes. In addition, SIRT3 knockout mice reportedly show more severe BBB disruption and inflammatory responses than do wild type mice in the acute phase, suggesting an underlying regulatory mechanism of HIF-1α/VEGF signaling in astrocytes and harmful effects in the acute phase of ischemic stroke (51). In general, reversal of HIF-1α expression in the acute phase may be a worthy target for ischemic stroke therapy. Edaravone has been shown to prevent the HIF-1α from binding to the VEGF promoter in hypoxic astrocytes, thereby maintaining the permeability of capillaries and venules after ischemic stroke. The regulatory effect on HIF-1α/VEGF signaling by Edaravone may partially contribute to its edema-resolving effects in acute ischemic stroke patients (52).

#### HIF-1α Regulates Glucose Flux From Hypoxia in Astrocytes

Astrocytes have been shown to transport glucose from the blood to provide energy to the brain parenchyma and maintain neuronal activity, a process in which HIF-1α is a key mediator (111, 112). As a vital link between oxygen stress and nutrient cellular adaptation, HIF-1α can enhance glycolytic flux by upregulating the expression of pivotal glycolytic genes, including *GLUT-1, phosphofructokinase 1, phosphoglycerate kinase-1, lactate dehydrogenase, and glucose-6-phosphate isomerase* (113, 114). GLUT-1 is expressed both in neurons and astrocytes primarily at the plasma membrane and is responsible for the entry of glucose into cells without consuming energy (115). HIF-1α is activated under hypoxic conditions, resulting in the transformation of GLUTs from the organelle membrane to the cytoplasmic membrane and the activation of glycolytic enzymes (53), promoting the uptake of glucose into cells and maintaining adenosine triphosphate (ATP) levels and cell survival. HIF-1α regulates glycometabolism through the glycolytic pathway to reduce ROS production in hypoxic environments. Research has revealed that activation of astrocyte mtCB1 receptors can lead to a reduction of ROS and impact the glycolytic production of lactate through the HIF-1 pathway, leading to neuronal redox stress and reduced cell viability in mouse astrocytes (54). Embryo fibroblasts in HIF-1α-knockout mice lose the ability of glycolytic metabolism during hypoxic conditions, resulting in cell death due to ROS overload (116). These findings demonstrated that HIF-1α regulates glucose flux to prevent increased ROS production under hypoxic conditions.

#### HIF-1α Protects Against Glutamate From Hypoxia in Astrocytes

It is known that astrocytes can rapidly eliminate neurotransmitters released in the synaptic cleft, such as glutamate (48). The release of glutamate and the influx of calcium ions are currently considered to be among the major pathological mechanisms in ischemic stroke. Further, HIF-1α protects against glutamate from hypoxia in astrocytes. One study showed that the elevation of HIF-1α improves cell viability in primary rat astrocytes under hypoxia and high concentrations of glutamate (0.1 and 1 mM for 3 h). Pretreatment with HIF-1α inhibitor was found to reduce the survival of astrocytes under treatment with 1 mM glutamate for 3 h, suggesting that HIF-1α provides protection against glutamate-induced excitatory toxicity in hypoxic astrocytes. Moreover, a correlation between increased glutathione and HIF-1α stabilization was observed in astrocytes (49).

#### HIF-1α and Neuroinflammation in Astrocytes

In addition to microglial cells, astrocytes have been shown to participate in both innate and subsequent adaptive immune responses (117). Moreover, they have been found to release chemokines, including CCL2, CCL20, and CXCL10, to attract neutrophils and monocytes into the hypoxic/ischemic brain, thus increasing neuroinflammation. HIF-1α was also found to mediate the transcriptional regulation of chemokines monocyte chemoattractant proteins 1 and 5 in hypoxic astrocytes, which aggravates the neuroinflammation injury after ischemic stroke (55). Interestingly, in the initial stages of ischemic stroke, astrocytes have been found to act as negative regulators of neuroinflammation by secreting transforming growth factor-β, interleukin (IL)-4, and IL-10. As ischemic injury progresses and the neuroinflammation becomes more severe, astrocytes release proinflammatory cytokines such as IL-1β, IL-6, and tumor necrosis factor (TNF)-α, which are HIF-1-dependent cytokines (118). Collectively, these findings indicate that HIF-1α plays a vital role in astrocyte-mediated immune response after stroke.

#### Other Signaling Pathways Mediated by HIF-1α in Astrocytes

In addition to mediation of the previously mentioned pathophysiological functions, HIF-1α may be involved in other signaling pathways related to neuroprotection. For instance, the STAT 3/HIF-1α/VEGF signaling pathway can be activated by the inhibition of Calpain-1 activity and consequent promotion of astrocytic neurogenesis, which improves stroke prognosis (119). Ischemic preconditioning, defined as transient exposure to subsequent prolonged cerebral ischemia, represents a fundamental adaptive response to environmental stress. A study found that hypoxia preconditioning induces the expression of P450 2C11, an arachidonic acid epoxygenase expressed in astrocytes, which was found to confer protection against ischemia-reperfusion damage through increased HIF-1α and to enhance the tolerance of astrocytes to OGD-induced hypoxic-ischemic injury *via* the P450 cyclooxygenase pathway (120). More recently, studies have focused on the formation of proteasome and their involvement in human disease (121, 122). Low molecular mass peptide 2 (LMP2), a subunit of the immunoproteasome (which is a proteasome subtype), has been found to participate in the inflammatory pathophysiological mechanisms of ischemic stroke in both experimental and clinical studies. Inhibition of LMP2 with shRNA has been reported to alleviate infarction and attenuate the expression of TNF-α and IL-1β (121, 123). In the OGD model of primary cultured astrocytes and the MCAO model, inhibition of LMP2 led to numerous HIF-1α neuroprotective effects, suggesting that HIF-1α may have a key role in the immunoproteasome-mediated inflammatory response (124). The above findings also imply that immunoproteasome inhibitors may constitute a promising strategy for stroke treatment.

### Microglia

Microglia are recognized as the resident phagocytes in the CNS and are major components of the innate immune response. As the final defense for brain parenchyma, microglia can identify subtle changes in the brain and immediately react to pathophysiological stimuli (125). As a result, activated microglia are recruited to the inflammatory site, where they eliminate toxins by phagocytosis, resolve inflammation, and repair tissue by producing anti-inflammatory factors such as IL-10 and transforming growth factor-β (TGF-β) to effectively maintain CNS homeostasis (126–128). However, microglia also promote inflammation after ischemia by secreting pro-inflammatory factors (ROS, TNF, and IL-1β). Once microglia are over-activated, uncontrolled excessive inflammation can cause host tissue damage, which is considered an essential factor leading to injury in the subacute phase of ischemic stroke.

#### Roles of HIF-1α in Microglia-Mediated Inflammation

The mRNA and protein levels of HIF-1a increase significantly in microglia after stimulation with pro-inflammatory cytokines TNF-α, IL-1, and IFN-γ, with the maximal induction occurring between 3 and 6 h (129). After ischemic stroke, HIF-1a facilitates the processes of microglia chemotaxis, phagocytosis, and ROS and TNF-α production by promoting the expression of the phagocytic genes *cd36* and *mfg-e8*, which have been shown to inhibit neurogenesis and lead to neuronal injury (130). Toll-like receptor 4 (TLR4) is a Type I transmembrane protein expressed in the surface of various immune cells, monocytes, macrophages, regulatory T cells, etc. Similarly, TLR4 has also been related to microglia-activation-mediated inflammatory responses in cerebral ischemia injury processes. Activation of TLR4 was found to promote microglia polarization and increase expression of proinflammatory factors, including TNF-α and IL-1β, ultimately aggravating neuronal damage (57). Under hypoxia, HIF-1α modulates the enhanced expression of TLR4 *via* increased phosphorylation of NF-κB in primary BV2 cell lines, thus exacerbating the inflammatory response. Inhibition of TLR4 with siRNA or antibodies was reported to reduce the expression of phospho-NF-κB and inflammatory factors TNF-α, IL-1β, and inducible nitric oxide synthase (iNOS) (58). The phosphatidylinositol 3-kinase (PI3K)/AKT/mammalian target of rapamycin (mTOR) pathway has been recognized as one of the upstream signaling pathways regulating the expression of HIF-1α (131). Salidroside, the bioactive component of *Rhodiola rosea* has shown neuroprotective effects, namely increasing the level of neuronal nuclear protein and reducing CD_11_b (a marker of microglia) through upregulation of the expression of HIF-1α and inhibition of the inflammatory response *via* the PI3K/AKT signaling pathway after cerebral ischemia in rats (132). In general, as the essential signaling regulator of inflammation, HIF-1α may be regulated by the PI3K/AKT pathway in brain tissue and participate in neuroinflammatory responses.

Therapeutic approaches targeting HIF-1α in microglia have shown neuroprotective effects. T-cell immunoglobulin and mucin domain protein-3 (TIM-3) are inhibitory molecules expressed on the T-cell surface. Research has indicated that glial TIM-3 is upregulated distinctively in activated microglia and astrocytes dependent on HIF-1 activation. Inhibition of TIM-3 has been found to substantially alleviate cerebral infarction, edema, neuronal cell death, and neutrophil infiltration in a mouse cerebral hypoxia-ischemia model, suggesting that TIM-3 is a modulator that links inflammation and subsequent brain damage after ischemia. Therefore, TIM-3 may serve as a downstream mediator of HIF-1α in the inflammatory response to hypoxic-ischemic damage (133). Arginine may protect against ischemic neuronal death after rat ischemia and reperfusion injury and inhibit inflammatory response by suppressing HIF-1α in OGD-cultured microglia and rat ischemic brain tissue (134). Glycine reduces neuroinflammation in rats with cerebral ischemia by downregulating NF-κB p65, thereby decreasing the expression of HIF-1α and ameliorating microglial polarization (135). Nucleotide-binding oligomerization domain, leucine-rich repeat and pyrin domain-containing 3 (NLRP3), one of the most studied inflammasomes (complexes of cytosolic proteins that identify pathogen signals), are predominantly activated by microglia under ischemic conditions (136). Once activated, inflammasomes produce IL-18 and IL-1β which are activated by caspase 1 and initiate the inflammatory response (137). Researchers have demonstrated that the NLRP3-dependent pyroptosis of cerebral cells is restrained by HIF-1α inhibition after 24 h of reperfusion following MCAO, indicating that HIF-1α may have a role in activating NLRP3 in microglia and mediating inflammatory responses (59).

Taken together, HIF-1a in microglia may serve as a potential therapeutic target for relieving inflammation reactions and neuronal damage after cerebral ischemia.

#### HIF-1α and Autophagy in Microglia

Autophagy is a process by which cytoplasmic material is degraded by lysosomes or vacuoles and then recycled to adapt to exogenous disruption (138). It is a vital process for tissue homeostasis that mediates and balances the favorable and adverse effects of inflammation. In microglia, autophagy appears to function mainly as an inhibitor of inflammatory responses (139, 140). Previous research has shown that deficiencies in autophagy caused by nutrient deprivation induce the activation and inflammation of microglia (140). More recently, researchers have found that HIF-1α may play a role in the autophagy-mediated activation of microglia (141). Under hypoxic conditions, the intensified autophagy appears to be mediated by the accumulation of HIF-1α in microglia, resulting in the inhibition of functions of autophagy-associated genes and the prevention of neuronal death in the initial stages. Moreover, genetic knockdown or pharmacological inhibition of HIF-1α was found to suppress autophagy and increase cellular viability (16). Another study confirmed that the inhibition of LC3-II (a direct indicator of autophagy) by HIF-1α induced the activation of autophagy in microglia (56). Although research has shown a correlation between HIF-1α and autophagy in microglia, the detailed mechanisms remain unclear.

### Pericytes

In addition to neurons, astrocytes, endothelial cells, and microglial cells, pericytes are an important component for maintenance of the vascular integrity of the NVU. However, few studies have focused on the role of HIF-1α in pericytes in the context of ischemic stroke. One study indicated that HIF-1 deficiency reduced ischemia-induced pericyte death, alleviated BBB permeability and preserved their coverage of the CNS microvasculature translating into a more stable barrier after transient MCAO in conditional mutant mice with pericyte-targeted HIF-1 loss of function (142). Further studies may focus on unveiling the biological function of HIF-1α in pericytes under pathological and physiological conditions, thus providing more favorable evidence on potential therapeutic targets for ischemic stroke. In addition to the abovementioned CNS cells, macrophages, neutrophils, dendritic cells, and lymphocytes are involved in the inflammatory response mediated by HIF-1α (143). However, more rigorous studies are needed to elucidate the specific roles of HIF-1α in infiltrated immune cells. HIF-1α is now considered a potential novel therapeutic target for ischemic stroke.

## Conclusion

In the treatment of acute ischemic stroke, intravenous thrombolysis with recombinant tissue plasminogen activator and mechanical thrombectomy have been shown to be extremely effective. These treatments, however, are still limited by their strict time frames. As a cellular sensor of oxygen level, HIF-1α is now considered as a potential novel therapeutic target for ischemic stroke and will hopefully serve as a predictor of stroke prognosis. An increasing number of new therapeutic strategies and agents focused on neuroprotection *via* regulation of HIF-1α are under development (**Table 2**), but these are yet to be tested in clinical trials. In the present review, we highlight the neuroprotective and detrimental effects of HIF-1α in in different cell types within the CNS under the context of stroke. We also summarize the representative therapeutic strategies based on the regulation of HIF-1α with the aim of providing guidance for future research on developing HIF-1α as a therapeutic target. However, differences in the functioning of HIF-1α between cell types and in different environments make it a challenging target in acute ischemic stroke. Simply activating or inhibiting the expression of HIF-1α for a therapeutic goal may lead to paradoxical results. Future studies are needed to focus on certain types of cells and bidirectional functions to gain a better understanding of HIF-1α in pre-clinical models of ischemic stroke before progressing to clinical trials. In conclusion, HIF-1α plays an important role in the aftermath of stroke and is a promising target for the treatment of ischemic stroke.

## Author Contributions

QH and YM designed and wrote the manuscript. JL, DZ, JR, RZ revise the manuscript. JC, Z-NG, and YY gave constructive advice and participated in proof-reading of this paper. All authors contributed to the article and approved the submitted version.

## Funding

This review was supported by the National Natural Science Foundation of China to YY (Grant No. 81771243), the Science and technology department of Jilin province (20180623052TC), and the Jilin Provincial Key Laboratory (20190901005JC) to YY.

## Conflict of Interest

The authors declare that the research was conducted in the absence of any commercial or financial relationships that could be construed as a potential conflict of interest.

## Publisher’s Note

All claims expressed in this article are solely those of the authors and do not necessarily represent those of their affiliated organizations, or those of the publisher, the editors and the reviewers. Any product that may be evaluated in this article, or claim that may be made by its manufacturer, is not guaranteed or endorsed by the publisher.
